# Is All Urban Green Space the Same? A Comparison of the Health Benefits of Trees and Grass in New York City

**DOI:** 10.3390/ijerph14111411

**Published:** 2017-11-18

**Authors:** Colleen E. Reid, Jane E. Clougherty, Jessie L.C. Shmool, Laura D. Kubzansky

**Affiliations:** 1 Department of Geography, University of Colorado Boulder, Boulder, CO 80309, USA; 2 Department of Environmental and Occupational Health, Dornsife School of Public Health, Drexel University, Philadelphia, PA 19104, USA; jcloughe@drexel.edu; 3 Department of Environmental and Occupational Health, Graduate School of Public Health, University of Pittsburgh, Pittsburgh, PA 15260, USA; jlcshmool@gmail.com; 4 Department of Social and Behavioral Sciences, Harvard T.H. Chan School of Public Health, Boston, MA 02115, USA; lkubzans@hsph.harvard.edu

**Keywords:** trees, grass, green space, self-reported health, socio-economic status

## Abstract

Living near vegetation, often called “green space” or “greenness”, has been associated with numerous health benefits. We hypothesized that the two key components of urban vegetation, trees and grass, may differentially affect health. We estimated the association between near-residence trees, grass, and total vegetation (from the 2010 High Resolution Land Cover dataset for New York City (NYC)) with self-reported health from a survey of NYC adults (n = 1281). We found higher reporting of “very good” or “excellent” health for respondents with the highest, compared to the lowest, quartiles of tree (RR = 1.23, 95% CI = 1.06–1.44) but not grass density (relative risk (RR) = 1.00, 95% CI = 0.86–1.17) within 1000 m buffers, adjusting for pertinent confounders. Significant positive associations between trees and self-reported health remained after adjustment for grass, whereas associations with grass remained non-significant. Adjustment for air pollutants increased beneficial associations between trees and self-reported health; adjustment for parks only partially attenuated these effects. Results were null or negative using a 300 m buffer. Findings imply that higher exposure to vegetation, particularly trees outside of parks, may be associated with better health. If replicated, this may suggest that urban street tree planting may improve population health.

## 1. Introduction

A growing literature documents numerous potential health benefits such as decreased mortality [1,2], better mental health [3,4], fewer adverse birth outcomes [5,6,7], and improved self-reported health [8,9,10] associated with living near vegetation, often called “green space” or “greenness” [4]. Most studies use vegetation indices from satellite imagery, such as the Normalized Difference Vegetation Index (NDVI), or combine all land cover types that are predominantly vegetation from land cover/land use datasets. For example, a number of studies have shown that better self-reported health is associated with levels of surrounding greenness [10], with higher percentages of green space near one’s home [9], and with objective measures of the quantity and quality of vegetation in a neighborhood [8]. These studies implicitly assume that total vegetation is what imparts health benefits. It is possible, however, that specific types of vegetation, such as trees and grass, differentially affect health.

Several studies have focused exclusively on exposure to trees, without comparison to grass, and they reported associations with increased physical activity [11], lower prevalence of obesity among preschoolers [12], lower prevalence of depression and stress among adults [13], lower rates of antidepressant prescriptions [14], better overall health [15], and lower risk of small-for-gestational-age birth [16]. Tree canopy cover has also been associated with higher rates of asthma and allergic sensitization, particularly to tree pollen among children growing up in New York City (NYC) [17]. Although proximity to grass could similarly increase allergic sensitization to pollen, we have identified no studies to date investigating this association. U.S. counties with declines in county-level tree coverage due to the emerald ash borer infestation have shown increased rates of lower respiratory and cardiovascular mortality [18] and cardiovascular disease onset [19].

A few studies have compared associations of trees versus grass with health, but notably, most of these studies have been done at coarse spatial resolution, such that it would be difficult to separate spatial distributions in trees and grass within those areas. For example, a study that focused only on undeveloped “natural” tree and grass areas—but did not assess trees and grass along streetscapes, in parks, or in private settings such as backyards—found a significant positive relationship between physical activity in children aged 11–13 and undeveloped treed areas, but not with undeveloped grassy meadows, within 1000 m of a child’s residence [20]. In an ecological analysis in the United Kingdom, Wheeler et al. (2015) [21] found benefits of grasslands and woodlands, based on land cover dataset definitions of these terms, on population general health. Another ecological study observed positive associations between forest edge density—a measure of ecological fragmentation rather than of specific types of vegetation—and area of herbaceous (grasslands) cover with physical activity at the county level in the U.S. [22]. Notably, all of these studies investigated treed or grassy areas at large spatial resolutions (e.g., county), and could not assess finer scale spatial variation in greenness (e.g., smaller areas of grass within a predominantly forested/woodland area, or trees within areas classified as grasslands/ grassy meadows/ herbaceous). Additionally, these studies did not focus on urban vegetation, the focus of our study, nor did they attempt to assess beneficial associations of vegetation separate from designated parklands, as we do. As many urban areas consider tree planting campaigns mostly along the streetscape, it is important to understand if trees and/or grass at smaller spatial scales benefit health differently. 

Researchers have proposed several pathways by which green space may affect health: psychological restoration and decreased psychological stress or distress [23,24], decreased air [25] and noise pollution [26], decreased ambient temperatures due to shade from trees and less impervious land cover [27], increased opportunities for physical activity [28] and social contacts [29], and increased exposure to beneficial microbiota [30]. Trees, as compared to grass, may play more of a role in providing shade, reducing noise pollution, or improving psychological restoration as has been shown in an experimental study [31].

Many studies hypothesize that air pollution is one mediating pathway between vegetation and health [32], however, some recent studies that have analyzed this pathway have not always found that this pathway plays a large role [1,6]. It is possible that the lower levels of air pollution found in greener areas is due to fewer sources of air pollution in greener urban areas rather than solely through trees absorbing air pollution. We, therefore, took a conservative approach to considering the role of air pollution (both NO_2_ and PM_2.5_) in the association of urban vegetation and self-reported health, hypothesizing that it might be acting as a confounder of the association between greenness and health.

Some studies have observed differential health effects of greenspace by socio-economic status (SES) [33,34]. These studies hypothesize that the greater benefits of greenness for lower SES groups could be due to lower health status in individuals with lower SES, such that incremental benefits are more observable, or because people with higher SES are more mobile and, therefore, greenness near home may not play as important a role in their health as it would in individuals with lower SES [33]. It is, therefore, plausible that any associations between health and living near trees and grass in an urban area could differ by SES. In addition, other research documents differential access to trees by race and income in various parts of the U.S. [35,36,37,38], and specifically in NYC, with lower-income neighborhoods having lower access to trees [39]. Thus, if health benefits accrue more to lower SES areas, this could be a further reason to focus urban greening efforts in those areas.

To our knowledge, our study is the first to compare observed associations with health for the two most prevalent urban vegetation types—trees and grass—separately and together in an urban setting. By combining a very finely-resolved (3-ft) land cover dataset for New York City (NYC) with data from a citywide health survey of NYC residents, we assessed whether trees and grass are independently associated with self-reported health, a measure shown to be a valid marker of overall health [40]. As many previous studies have examined associations between urban parks (rather than total greenspace areas) and health [41], we also assessed whether relationships between trees or grass and self-reported health were independent of living near parks or open spaces. We further investigated whether SES would modify the relationships between each vegetation type and self-reported health. Determining more concretely what health benefits might accrue to specific populations with each type of vegetation exposure could prove important as other cities look to inform efforts to increase urban forests.

## 2. Materials and Methods

### 2.1. Study Population

Our study population includes 1549 residents of NYC who responded to a survey about neighborhood stressors, psychosocial stress, and health. The survey was conducted in summer (June–September 2012) and winter (December–March 2012–2013) through random digit dialing (RDD) of landlines and cell phones and an online survey. The RDD surveys were implemented by trained administrators at the Survey Research Program of the University Center for Social and Urban Research at the University of Pittsburgh. Administrators assessed the eligibility of participants, and obtained informed consent before survey responses were collected. The online survey was self-administered among participants of a voluntary, standing survey panel of NYC adults (Survey Sampling International, http://www.surveysampling.com, MyOpinions Pty Ltd., Shelton, CT, USA). 

Survey participants were spatially-distributed across all NYC boroughs, approximately proportional to borough populations. Our rates are comparable with national average response, cooperation, and contact rates [42]. Response rate for RDD landline and cellular sampling frames were low (5.5% out of approximately 12,500 contacted), reflecting low contact (38.8% summer; 39.9% winter) and cooperation rates (14.8% summer; 13.1% winter). Across seasons, sample participants were drawn approximately 34% from RDD landline, 10% from RDD cellular, and 55% from online frames. Compared to NYC census statistics (American Community Survey 2008–2012), the survey under-represented individuals aged 45–65 years, those with less than high school education, and males. It over-represented low-income households (annual income < Federal Poverty Level (FPL)). Survey protocols were approved by the University of Pittsburgh Institutional Review Board and analysis of the data was approved by the Institutional Review Boards of the University of Pittsburgh, Harvard T.H. Chan School of Public Health, and the University of Colorado Boulder. 

Survey respondents provided their nearest cross-streets rather than actual address for confidentiality concerns. In NYC, the nearest cross-street would most often be within 174 m (i.e., half of a standard Manhattan block length). Reported nearest cross-streets of 1,439 survey participants were successfully geocoded. Of those, 52 were removed because their reported nearest cross-streets did not geocode to a habitable area within NYC: 29 geocoded to water areas, 21 to areas within a large city park, and two to areas outside of NYC. Of the remaining 1387 individuals, 106 had missing values for one or more covariates. All analyses were based on the 1,281 individuals with complete information. Those removed for this analysis were not significantly different from those retained in sex, age, or income, but had lower educational attainment, on average (Table S1). 

### 2.2. Exposure Assessment

We used the NYC high-resolution (3-ft) Landcover Raster Data from 2010 (https://data.cityofnewyork.us/Environment/Landcover-Raster-Data-2010-/9auy-76zt) to estimate exposure to trees and grass, two of seven land cover classifications (tree canopy, grass/shrubs, bare earth, water, buildings, roads, and other paved surfaces) in the dataset. The data are publicly available and were created from 2010 LiDAR (Light Detection and Ranging) data and 2008 4-band orthoimagery, with a per-pixel accuracy of 96% [43]. Any ground area covered by tree canopy was classified as tree-covered. A map of the tree and grass coverage for NYC from this dataset as well as a map of survey respondent locations are provided in Figure S1. 

Each participant was assigned exposure to trees, grass, and total vegetation (trees + grass) as the percent of each within 300 m and 1000 m radial buffers around their self-reported nearest cross-street. These buffer sizes were chosen in keeping with previously-used and sensitivity-tested methods for air pollution exposure assessment in NYC [44].

### 2.3. Outcome Assessment

The self-reported health measure was a single validated item drawn from the NYC Department of Health and Mental Hygiene Community Health Survey [45], previously found to predict mortality when used either as a dichotomous or as a continuous measure [40,46]. Survey respondents were asked: “Would you say that in general your health is excellent, very good, good, fair or poor”? Following prior work considering effects of greenspace on health which have considered this measure as a dichotomous measure [47,48], we dichotomized responses into a category of “very good” or “excellent” health (1) compared to “good”, “fair”, or “poor” health (0). In a sensitivity analysis, we also considered self-reported health as a continuous outcome, following previous work [8]. 

### 2.4. Covariate Assessment

From the survey, we extracted each individual’s self-reported age, income category (by multiples of the FPL), educational level, season of survey (i.e., winter or summer), sampling frame (i.e., landline RDD, cellular phone RDD, online panel), and neighborhood tenure. 

To obtain a comprehensive measure of SES, we created a composite measure combining dichotomized measures of individual-level income and education to create four mutually-exclusive categories with sufficient numbers of survey respondents in each category. Researchers often assess SES in a variety of ways, often using education and income, either as a combined measure or separately [49,50]. Our study sample had a higher proportion of individuals with high education but lower income than expected. As a result, we made the choice to combine these measures and investigate potential effect modification by the combined measure. This yielded n = 427 people with both higher income (≥$46,000 annual income, which was twice the FPL for a family of four in 2012) and higher education (Bachelor’s degree or higher), n = 171 people with higher education but lower income (<$46,000 annual income), n = 238 people with lower education (less than a Bachelor’s degree) but higher income, and n = 445 people with lower education and lower income. 

We obtained, at the census tract-level, variables (2010 Census area designations) for area-level population density and SES (% living below 200% of the FPL and % unemployed) from the five-year running average of the American Community Survey for the years 2008–2012.

Air pollution exposure estimates for NO_2_ and PM_2.5_ were derived from two-year surfaces from the New York City Community Air Survey (NYCCAS) (2008–2010) [51], an on-going air pollution surveillance initiative of the New York City Department of Health and Mental Hygiene; monitoring and modeling methods are detailed elsewhere [52,53]. Briefly, two-week integrated street-level samples were collected at 150 monitoring locations across NYC. Land Use Regression was used to predict fine-scale concentration estimates corresponding to 300 m grid centroids, enabling fine-scale exposure estimates. We estimated pollution exposures for each respondent from these surfaces within the same radial buffers as for vegetation.

We calculated the percent city-designated park area using the NYC Department of Parks and Recreation Parks Properties shapefile (https://data.cityofnewyork.us/City-Government/Parks-Properties/rjaj-zgq7), and percent of non-park open space using the NYC Open Space (not parks) shapefile (https://data.cityofnewyork.us/Recreation/Open-Space-Other-/pckb-8r2z) within each radial buffer. We note that both parks and open space may contain both green and non-green areas. 

### 2.5. Statistical Analysis

Our binary outcome was not rare (47.2% of respondents reported “very good” or “excellent” health), therefore, we used generalized estimating equations (GEE) Poisson regression models to estimate associations between percent trees and grass density, separately and together, with dichotomized self-reported health. Vegetation exposure variables were modeled as quartiles to assess potentially non-linear associations. We first ran unadjusted models, then in our second model adjusted for age, sex, race/ethnicity, sampling frame, season, neighborhood tenure, individual-level SES (income, educational attainment), and area-level SES (% living below twice the FPL and % unemployed at the census tract). Age, % living below twice the FPL, and % unemployed were modeled as continuous variables; all others as categorical. In a third model, we also adjusted for nitrogen dioxide (NO_2_). The fourth model further adjusted for percent park and non-park open spaces. We used the Huber-White sandwich estimator to adjust properly for clustered responses at the census tract and obtain robust standard errors.

To assess possible effect modification by SES, we created interaction terms by multiplying each comprehensive SES category by percent trees and grass separately. These models also adjusted for individual and area-level covariates, and further models adjusted for NO_2_ and parks. 

We performed several sensitivity analyses; we considered self-reported health as a continuous variable using standard linear regression adjusted for all covariates and assessed greenspace as a continuous measure rather than in quartiles. We also explored adjustment for PM_2.5_ instead of, and in addition to, NO_2_ as well as population density instead of air pollution. We also assessed effects of estimating exposures at different buffer sizes (100 m, 500 m, 2000 m). All analyses were done in 2015–2016 in R version 3.4.0 (R Core Team, Vienna, Austria). 

## 3. Results

Near-residence tree and grass cover varied substantially across our cohort. On average, 18.1% (range 1.5–55.1%) of the 1000 m or 18.2% (range 1.1–64.9%) of the 300 m radius around the geocoded nearest cross-streets was covered by trees; only 10.3% (range 0.9–40.5%) (1000 m) and 9.2% (range 0.0–58.0%) (300 m) was covered by grass (Table 1). Of the 1,281 survey respondents included in our analysis, 185 responded that their self-reported health was excellent, 420 as “very good”, 439 as “good”, 198 as “fair”, and 39 as “poor”. Table 2 details the distribution of covariates by quartiles of percent trees and grass, by buffer size.

Percent trees and grass were correlated at 1000 m (r = 0.46) and 300 m (r = 0.41). As expected, percent grass [r = −0.71 (1000 m), r = −0.66 (300 m)] and trees [r = −0.44 (1000 m), r = −0.66 (300 m)] were strongly negatively correlated with NO_2_. However, NO_2_ was weakly positively correlated with income [r = 0.04 (1000 m), r = 0.05 (300 m)] and education [r = 0.12 (1000 m), r = 0.13 (300 m)], consistent with findings from other NYC studies [54]. 

Higher tree density within 1000 m was associated with higher likelihood of reporting “very good” or “excellent” health (comparing the highest quartile to the lowest quartile relative risk (RR) = 1.23, 95% CI = (1.06, 1.44) adjusted for individual-level and area-level SES (Table 3). At the 300 m buffer, however, we observed no associations between trees and self-reported health. Grass at the 1000 m buffer was not associated with self-reported health, but at the 300 m buffer we observed an apparent negative association between percent grass and self-reported very good or excellent health. The associations between total vegetation and self-reported health were intermediate of the associations found for the two vegetation types (Table 3). 

Associations between exposure to any vegetation type and self-reported health became slightly more positive after additional adjustment for NO_2_, becoming significantly positive in the case of grass within 1000 m buffers. Further adjustment for percent parks and open spaces had minimal effect on results for both trees and grass at each buffer size (Table 3), suggesting that trees and grass outside of parks, such as street trees and grassy medians, influence people’s self-reported health. Tests for trend were only significant for total vegetation at the 1000 m buffer when adjusted for SES and NO_2_, or for SES, NO_2_, and parks (Table 3). Results were similar after adjusting for PM_2.5_, instead of, or in addition to, NO_2_, and for adjustment for population density (Table S2). 

In models containing a percentage of both grass and trees simultaneously, associations observed were consistent with models including only one vegetation type (Table 4). 

**Figure 1:**
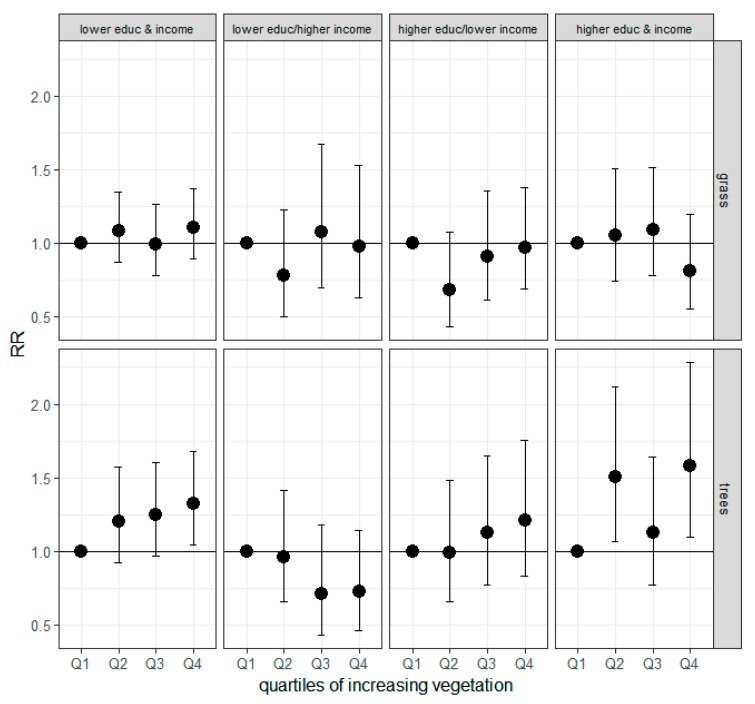
RRs for self-reported “very good” or “excellent” health by quartiles of vegetation density within the 1000 m buffer and SES categories. Models were additionally controlled for season, sampling frame, neighborhood tenure, age, sex, race/ethnicity, individual SES (two-level categorical variable for education, and a six-level categorical variable for income), and area-level SES (% of census tract unemployed and % of census tract living below twice the FPL), RR = relative risk.

At the 1000 m buffer, higher tree density was associated with better self-reported health among individuals with higher income/higher education and with lower income/lower education, but not for individuals with higher income/lower education or lower income/higher education. Higher grass density, however, was not associated with better self-reported health in any SES category (Figure 1). Further adjustment for NO_2_ slightly enhanced RRs such that increasing grass density was associated with better health among only low income/low education individuals (Figure S2). There was no apparent association between trees or grass within the 300 m buffers in self-reported health for any SES category.

Considering self-reported health as a continuous variable (Table S3) or percent vegetation as a continuous predictor, rather than by quartiles (Table S4), did not alter our findings. 

We found no evidence of spatial clustering of the residuals from our main finding (with percent of trees within a 1000 m buffer, after adjusting for SES, area SES, and NO_2_) using Moran’s I (with conceptualizations of neighboring using both inverse distance (I = 0.0070, *p* = 0.83), inverse distance squared (I = 0.0072, *p* = 0.82), and fixed distance cutoffs of 1000 m (I = 0.0045, *p* = 0.68), 500 m (I = 0.03, *p* = 0.15), and 300 m (I = 0.014, *p* = 0 .57)).

## 4. Discussion

This is the first study, to our knowledge, to compare the associations for trees versus grass with self-reported health in an urban setting. Previous work on green space and health has generally examined total vegetation, rather than separating it into various types, and those that do investigate different types have quantified these vegetation types at much coarser spatial scales. We found some evidence of differences whereby beneficial health associations of trees were somewhat stronger and more consistent than those for grass. In addition, exposure to trees (and, to a lesser extent, grass) showed positive associations with better self-reported health when holding exposure to parks and open spaces constant.

Findings, however, were not uniform across buffer sizes. All significant positive associations were found only with 1000-m buffers. A recent review of greenness studies found that while some studies found the benefits of greenness at small buffer sizes, others found significant benefits of vegetation within further distances from the location of residence [55]. This could be specific to the outcome being studied, the hypothesized pathway by which the effect occurs, or the context or location of the study. Within studies investigating the effect of residence-area greenness on self-reported health with different buffer sizes, Dadvand et al. (2016) found consistent associations using 100, 250, and 500 m buffers [48], Orban et al. (2017) found consistent associations at 100 and 1000 m buffers [56], and de Vries et al. (2003) found significant positive associations at 1000 and 3000 m, but stronger associations with greenspace at further distances (between 1000–3000 m) [47]. Therefore, our significant findings at 1000 m are consistent with previous work, but our null findings at 300 m are not [9,47]. Sensitivity analyses with other buffer sizes continued to suggest that vegetation in larger buffers was more strongly associated with self-reported health in our study (Table S5). Different findings by buffer size are likely influenced by how much greenspace varies across the study area. Therefore, differential findings by buffer size in our study could be due to high spatial variability in tree and grass coverage in NYC versus other locations; findings may be different in regions with more uniform greenness. Also, in a dense city such as NYC, we found greater densities of trees than grass for most survey respondents, at each buffer size. For example, median percent area covered by trees at the 300-m buffer was 17.0, and at 1000 m was 16.9, whereas it was 7.21 and 8.40, respectively, for grass. Differences in findings that are due to the spatial unit used for contextual variables is referred to as the uncertain geographic context problem [57], and the optimal spatial unit is often unknown [58]. Buffers may not accurately represent exposures, as many individuals do not spend the majority of their time within these buffers. Another concern in neighborhoods research is the ‘local trap’ in which many studies fail to recognize that people’s perceived and experienced neighborhoods are often much larger than researchers may expect [59]. It is possible that larger buffer sizes better represent where people actually spend their time than do smaller spaces. Given these issues, studies of green space and health should evaluate effects at multiple buffer sizes whenever possible. 

Because trees and grass tend to be spatially correlated, a study examining only one may actually reflect effects of both vegetation types. Across our cohort, however, the correlation between trees and grass was only moderate (Pearson’s r = 0.47 and 0.41, at the 1000- and 300-m buffers, respectively) making it appropriate to consider them separately. Our differential findings for trees and grass could mean that these vegetation types act through different hypothesized pathways towards better health. Trees, for example, may be more likely than grass to provide shade or reduce noise pollution. Trees also release terpenes, many of which demonstrate anti-inflammatory, anti-tumorigenic, and neuroprotective effects in toxicological studies [60]. Trees have also been shown to be associated with other benefits such as reduced crime [61,62], which may be related to psychological pathways to health. More research is needed to determine which pathways are most influential for specific health outcomes and for specific vegetation types.

Another important finding from our research was that adjusting for parks only slightly attenuated the positive association between trees and health, implying that non-park trees (i.e., street trees) may benefit health separately from park trees in NYC. Other studies have documented benefits of streetscape greenery on self-reported health, although they did not differentiate the streetscape vegetation into types, such as trees or grass [8].

Despite previous studies hypothesizing that health benefits of green space are partly due to air pollution removal [4], we found adjustment for air pollutants resulted in stronger associations, suggesting that air pollution acted as a negative confounder. While these findings may be specific to NYC, previous studies of green space and health elsewhere have found that air pollutants explain little [1] to no [6] portion of the relationship between green space and health. Although there is some evidence that air pollutants are filtered by trees [63], there is also evidence that this may be a small effect [64], and that the negative correlation between green space and air pollution may actually be driven by an absence of pollution sources (i.e., vehicles, industry) in that specific location, as has been previously hypothesized in NYC [65]. 

Our current study found differential associations of trees and grass with self-reported health across SES groups. Trees were associated with higher rates of “very good” or “excellent” health in both higher-SES and lower-SES individuals, although the benefits appeared stronger among the higher-SES individuals. Grass was only associated with self-reported health with adjustment for NO_2_, and only for low-SES individuals. This variation in findings by SES and type of vegetation could be due to differential perceptions of safety in greener areas [66], connectedness to nature [67], or quality of vegetation [68], as recent studies have found these factors also modify associations between greenness and health and should be further studied to determine whether they are related to SES. Previous investigations of effect modification by SES in studies of green space and health have generally found stronger beneficial associations for individuals with lower versus higher educational attainment [9,33,69,70], in areas with greater income deprivation [71], and among those with greater perceived financial strain [72]. A number of other studies, however, have not found differential associations by SES [10,73]. Inconsistent findings may be due to a number of methodological issues or because studies are using a diverse set of health endpoints. Interestingly, all studies examining this issue to date were conducted in European contexts where the relationships between green space, SES, and health may differ from the U.S. Further research is needed to better understand these pathways, and others, which may modify effects across population subgroups. 

Although efforts were made to recruit a sample of survey respondents representative of the population of NYC, our sample under-represented individuals aged 45–65, those with less than high school education, and males. It over-represented low-income households (annual income < FPL). Although age and education may be associated with both health and greenness, we did adjust for these variables in our models. However, there is the potential for residual confounding. Though all final models were adjusted for sampling frame (selected through landline RDD, cell phone RDD, or online), non-random recruitment of the online sampling panel may limit generalizability of our findings, despite a comparable spatial distribution and demographic composition compared to participants recruited through the RDD frames. There is also the possibility of selection bias if survey respondents were not representative of variation in neighborhood vegetation and self-reported health. The survey was originally written to understand perceptions of chronic stress and other neighborhood conditions, and did not include questions on vegetation. It is possible, though unlikely, that survey respondents were more likely to live in areas of greater tree coverage and have better health, or vice-versa, than is the norm. Although our response, contact, and cooperation rates were within range of national averages [42], they were still low, and some unmeasured bias is possible. Because analyses were cross-sectional, we cannot infer a causal relationship between vegetation exposures and self-reported health. Additionally, we did not have information on quality of vegetation nor species type of trees or grasses; further research should investigate these variables with health. 

There is a temporal mismatch between our exposure data (2010) and the survey data (2012–2013). During this time, NYC was undergoing a Million Street Trees campaign, which was started in 2007 and was half-completed by the fall of 2011. Therefore, more trees were added between the time when the exposure data was created and the time at which we administered the survey. These newly-added trees, however, were likely to be small saplings at the time of the survey, and, therefore, depending on the pathway by which health benefits may accrue, may not have provided many of the hypothesized benefits (e.g., shading/cooling, visual benefits through psychological restoration) as would older, larger trees. Thus, it seems less likely that changes in tree cover over time would substantially alter our basic findings. In fact, if the tree plantings were spatially distributed in a way that was unrelated to self-reported health, this would have likely made our findings underestimates of the true relationship between trees and self-reported health. However, longitudinal analysis of the health associations with the rollout of the NYC Million Trees Campaign would greatly enhance our understanding of the potential health benefits of trees. 

Our study also has a number of strengths, including a very fine-resolution (3-ft) land cover dataset, which enabled distinction between trees and grass, and individual-level information on health status and a range of potential confounders. Our results are consistent with previous studies documenting health benefits of trees [11,12,16,18,19], but extend these findings by suggesting that trees may more strongly benefit health than does grass in an urban setting.

## 5. Conclusions

Findings from the present study may inform methods for future research, specifically regarding buffer size, and vegetation types. Future work will require careful attention to the particular location under study, recognizing that effects may be context-dependent, at both metropolitan and neighborhood scales. Should findings from the present study be replicated in other contexts and with other health outcomes, however, they may suggest that cities could improve resident health by planting trees, a relatively affordable and simple intervention. These health benefits would be in addition to the numerous environmental benefits of urban tree planting related to mitigating, through sequestering carbon dioxide and reducing the need to cool buildings [74], and adapting to, through reducing the urban heat island effect [75], climate change.

## Figures and Tables

**Table 1:** Descriptive statistics.

Variables	Mean (Range) or %
*Exposure Variables:*	
% Trees (1000 m)	18.1 (1.5–55.1)
% Trees (300 m)	18.2 (1.1–64.9)
% Grass (1000 m)	10.3 (0.9–40.5)
% Grass (300 m)	9.2 (0.0–57.9)
% Total vegetation (1000 m)	28.3 (4.9–76.9)
% Total vegetation (300 m)	27.4 (1.3–91.5)
*Outcome Variables:*	
Self-reported health	
“Excellent”	14.4
“Very good”	32.8
“Good”	34.3
“Fair”	15.5
“Poor”	3.0
*Covariates:*	
Age	44.7 (18–90)
Sex	
Male	36.4
Female	63.6
Ethnicity	
Hispanic	20.3
Non-Hispanic	79.7
Race	
White	54.6
Non-White	45.4
Educational Level	
Less than high school	4.3
High school graduate to less than BA	49.0
BA or higher	46.7
Income	
<$23,000	24.3
$23,000 to <$46,000	23.8
$46,000 to <$70,000	18.7
$70,000 to <$93,000	13.2
$93,000 to <$135,000	10.1
≥$135,000	10.0
How long lived in the neighborhood	
<1 year	6.4
1 to <5 years	20.1
5 to <10 years	15.1
10+ years	58.4
Survey frame	
Cell phone	9.4
Landline	32.0
Online	58.6
% Census tract unemployed	9.7 (0.0–100.0)
% of Census tract living below twice the FPL	34.7 (0.0–100.0)
NO_2_ within 300 m (ppb)	25.4 (12.6–51.4)
NO_2_ within 1000 m (ppb)	24.8 (13.9–46.8)

BA: bachelor’s degree, FPL: Federal Poverty Level.

**Table 2:** Covariate distribution (n) from N = 1281 across quartiles (Q) of greenspace.

Covariate	% Trees—300 m Buffer				% Trees—1000 m Buffer				% Grass—300 m Buffer				% Grass—1000 m Buffer			
Quartile	Q1	Q2	Q3	Q4	Q1	Q2	Q3	Q4	Q1	Q2	Q3	Q4	Q1	Q2	Q3	Q4
Range of exposure value	(1.14, 12.6)	(12.6, 17.0)	(17.0, 21.9)	(21.9, 64.9)	(1.47, 13.3)	(13.3, 16.9)	(16.9, 21.5)	(21.5, 55.1)	(0.04, 3.72)	(3.72, 7.22)	(7.22, 13.09)	(13.09, 57.95)	(0.89, 5.71)	(5.71, 8.40)	(8.40, 14.33)	(14.33, 40.54)
Self-reported health status																
Very good or excellent	149	141	162	153	132	154	144	175	177	133	137	158	155	142	153	155
Good, poor, or very poor	170	180	158	168	189	167	175	145	144	188	180	164	166	177	169	164
Sex																
Male	133	102	109	122	116	125	119	106	120	99	125	122	117	104	128	117
Female	186	219	211	199	205	196	200	214	201	222	192	200	204	215	194	202
Education																
Less than HS	15	17	12	11	17	20	16	2	15	15	18	7	14	12	21	8
>HS but <BA	163	161	159	145	156	166	167	139	120	157	163	188	116	162	174	176
BA or higher	141	143	149	165	148	135	136	179	186	149	136	127	191	145	127	135
Income categories																
<$23,000	84	85	75	67	81	95	86	49	69	95	87	60	72	94	89	56
$23,000 to <$46,000	78	73	87	67	73	80	78	74	67	83	73	82	70	70	85	80
$46,000 to <$70,000	60	66	55	58	62	49	67	61	59	46	62	72	47	61	62	69
$70,000 to <$93,000	43	40	40	46	35	47	35	52	48	38	36	47	51	39	31	48
$93,000 to <$135,000	30	30	28	42	39	26	19	46	31	29	31	39	35	27	31	37
$135,000+	24	27	35	41	31	24	34	38	47	30	28	22	46	28	24	29
Race																
Non-White	152	160	146	124	142	173	160	107	116	168	165	133	111	177	175	119
White	167	161	174	197	179	148	159	213	205	153	152	189	210	142	147	200
Ethnicity																
Non-Hispanic	234	259	263	265	243	251	261	266	257	255	239	270	262	236	249	274
Hispanic	85	62	57	56	78	70	58	54	64	66	78	52	59	83	73	45
How many years lived in neighborhood																
<1	26	22	22	12	18	16	26	22	25	18	24	15	19	23	22	18
1 to <5	70	61	62	65	69	65	68	56	65	82	58	53	71	78	53	56
5 to <10	53	45	52	43	47	45	49	52	50	40	41	62	39	46	56	52
10+	170	193	184	201	187	195	176	190	181	181	194	192	192	172	191	193
Season																
Summer (June–September 2012)	143	157	173	149	146	152	151	173	161	152	143	166	156	142	164	160
Winter (December 2012–March 2013)	176	164	147	172	175	169	168	147	160	169	174	156	165	177	158	159
Sampling frame																
Cell	31	35	35	20	29	36	32	24	24	27	35	35	19	34	35	33
Landline	81	91	105	133	102	102	101	105	89	108	108	105	103	87	121	99
Online	207	195	180	168	190	183	186	191	208	186	174	182	199	198	166	187
Age category																
18–24	53	38	52	26	41	43	48	37	39	47	39	44	36	44	47	42
25–34	76	75	71	59	75	74	68	64	83	65	67	66	70	82	66	63
35–44	60	45	40	52	52	46	51	48	41	57	487	52	54	40	42	61
45–54	60	53	53	62	53	48	63	64	51	61	60	56	46	63	62	57
55–64	45	66	52	61	51	70	48	55	64	49	62	49	60	54	59	51
65+	25	44	52	61	49	40	41	52	43	42	42	55	55	36	46	45
Composite SES																
High education and income	101	101	103	122	104	90	100	133	138	94	102	93	133	104	91	99
High education/low income	40	42	46	43	44	45	36	46	48	55	34	34	58	41	36	36
Low education/high income	56	62	55	65	63	56	55	64	47	49	55	87	46	51	57	84
Low education and income	122	116	116	91	110	130	128	77	88	123	126	108	84	123	138	100

HS = high school. BA = bachelor’s degree; SES = socio-economic status.

**Table 3:** Relative risks (RRs) for self-reported “very good” or “excellent” health by quartile of vegetation density.

Buffer	Exposure	Covariates	Q1 (Reference)	Q2	Q3	Q4
1000 m buffer	Trees	unadjusted	1	1.17 (0.99, 1.38)	1.11 (0.93, 1.33)	1.33 (1.14, 1.56) *
		SES	1	1.20 (1.02, 1.41) *	1.10 (0.93, 1.31)	1.23 (1.06, 1.44) *
		SES + NO_2_	1	1.24 (1.05, 1.46) *	1.17 (0.98, 1.39)	1.37 (1.15, 1.63) *
		SES + NO_2_ + parks	1	1.24 (1.05, 1.46) *	1.16 (0.98, 1.39)	1.36 (1.13, 1.64) *
	Grass	unadjusted	1	0.93 (0.78, 1.11)	0.98 (0.84, 1.15)	1.00 (0.86, 1.18)
		SES	1	0.99 (0.84, 1.17)	1.05 (0.90, 1.23)	1.00 (0.86, 1.17)
		SES + NO_2_	1	1.09 (0.91, 1.30)	1.23 (1.00, 1.50) *	1.25 (1.00, 1.57) *
		SES + NO_2_ + parks	1	1.07 (0.89, 1.28)	1.21 (0.98, 1.48)	1.21 (0.96, 1.53)
	Total Vegetation	unadjusted	1	0.91 (0.76, 1.08)	1.09 (0.93, 1.28)	1.16 (1.00, 1.35) *
		SES	1	1.00 (0.84, 1.18)	1.11 (0.95, 1.30)	1.12 (0.96, 1.30)
		SES + NO_2_^**^	1	1.09 (0.91, 1.31)	1.27 (1.07, 1.52) *	1.42 (1.15, 1.74) *
		SES + NO_2_ + parks ^**^	1	1.09 (0.91, 1.32)	1.27 (1.06, 1.53) *	1.42 (1.13, 1.77) *
300 m buffer	Trees	unadjusted	1	0.95 (0.80, 1.14)	1.08 (0.93, 1.26)	1.02 (0.87, 1.20)
		SES	1	0.96 (0.81, 1.14)	1.06 (0.91, 1.23)	0.98 (0.83, 1.15)
		SES + NO_2_	1	0.99 (0.83, 1.17)	1.10 (0.94, 1.29)	1.05 (0.88, 1.26)
		SES + NO_2_ + parks	1	0.99 (0.84, 1.18)	1.11 (0.95, 1.30)	1.09 (0.91, 1.31)
	Grass	unadjusted	1	0.76 (0.64, 0.89)	0.78 (0.67, 0.92)	0.89 (0.77, 1.03)
		SES	1	0.81 (0.69, 0.96)	0.81 (0.69, 0.94)	0.91 (0.78, 1.05)
		SES + NO_2_	1	0.84 (0.70, 1.01)	0.85 (0.71, 1.03)	0.98 (0.80, 1.22)
		SES + NO_2_ + parks	1	0.84 (0.70, 1.01)	0.86 (0.71, 1.04)	0.98 (0.79, 1.22)
	All Vegetation	unadjusted	1	0.85 (0.72, 1.01)	0.94 (0.80, 1.10)	0.95 (0.81, 1.11)
		SES	1	0.86 (0.73, 1.02)	0.92 (0.79, 1.07)	0.90 (0.77, 1.06)
		SES + NO_2_	1	0.89 (0.75, 1.06)	0.98 (0.82, 1.18)	1.01 (0.82, 1.24)
		SES + NO_2_ + parks	1	0.90 (0.76, 1.07)	0.99 (0.83, 1.19)	1.03 (0.83, 1.28)

All models also controlled for season, sampling frame, neighborhood tenure, age, sex, race/ethnicity, individual SES (two-level categorical variable for education, and a six-level categorical variable for income), and area-level SES (% of census tract unemployed and % of census tract living below twice the Federal Poverty Level (FPL)). Adjustment for park spaceused percent of area within each buffer comprised of a city park or other open space. Results were similar adjusting for parks alone or for both parks and other open spaces. SES: socio-economic status, * *p* < 0.05, ** test for trend *p* < 0.05.

**Table 4:** RRs for self-reported “very good” or “excellent” health by quartiles of simultaneously-adjusted trees or grass.

Exposure	Covariates	Q1 (Reference)	Q2	Q3	Q4
Trees (1000 m buffer)	Grass	1	1.21 (1.02, 1.44) *	1.18 (0.98, 1.42)	1.44 (1.21, 1.72) *
	Grass + SES	1	1.22 (1.04, 1.44) *	1.14 (0.96, 1.37)	1.30 (1.09, 1.55) *
	Grass + SES + NO_2_	1	1.23 (1.04, 1.45) *	1.15 (0.96, 1.38)	1.33 (1.11, 1.60) *
	Grass + SES + NO_2_ + parks	1	1.22 (1.04, 1.44) *	1.15 (0.96, 1.37)	1.33 (1.10, 1.61) *
Grass (1000 m buffer)	Trees	1	0.86 (0.72, 1.02)	0.88 (0.75, 1.04)	0.85 (0.71, 1.01)
	Trees + SES	1	0.92 (0.78, 1.09)	0.96 (0.81, 1.14)	0.90 (0.75, 1.07)
	Trees + SES + NO_2_	1	1.01 (0.84, 1.22)	1.12 (0.91, 1.38)	1.13 (0.89, 1.42)
	Trees + SES + NO_2_ + parks	1	1.01 (0.84, 1.22)	1.12 (0.91, 1.38)	1.13 (0.89, 1.42)
Trees (300 m buffer)	Grass	1	1.00 (0.84, 1.20)	1.18 (1.00, 1.39) *	1.14 (0.95, 1.36)
	Grass + SES	1	1.01 (0.85, 1.20)	1.14 (0.97, 1.34)	1.08 (0.90, 1.29)
	Grass + SES + NO_2_	1	1.01 (0.85, 1.20)	1.14 (0.97, 1.34)	1.09 (0.91, 1.31)
	Grass + SES + NO_2_ + Parks	1	1.01 (0.85, 1.21)	1.15 (0.98, 1.35)	1.13 (0.94, 1.36)
Grass (300 m buffer)	Trees	1	0.73 (0.62, 0.86) *	0.74 (0.63, 0.88) *	0.83 (0.70, 0.97) *
	Trees + SES	1	0.79 (0.67, 0.94) *	0.78 (0.66, 0.92) *	0.87 (0.74, 1.02)
	Trees + SES + NO_2_	1	0.82 (0.69, 0.98) *	0.82 (0.68, 1.00)	0.94 (0.76, 1.17)
	Trees + SES + NO_2_ + Parks	1	0.82 (0.68, 0.98) *	0.83 (0.68, 1.00)	0.95 (0.77, 1.18)

All models controlled for season, sampling frame, neighborhood tenure, age, sex, race/ethnicity, individual SES (two-level categorical variable for education, and a six-level categorical variable for income), and area-level SES (% of census tract unemployed and % of census tract living below twice the FPL). Adjustment for park space used percent of area within each buffer comprised of a city park or other open space. Results were similar adjusting for parks alone or for both parks and other open spaces. * *p* < 0.05

## Supplementary Materials

The following are available online at www.mdpi.com/1660-4601/14/11/1411/s1, Figure S1: Map of tree and grass coverage at 3-ft resolution for all of NYC, Figure S2: RRs for self-reported very good or excellent health by quartiles of % grass or % trees at 1000m buffer by categorical SES levels additionally adjusted for individual-level and area-level SES, and NO_2_, Table S1: Comparison of survey sample retained in analysis to those omitted due to non-geocodeable residential cross-streets, Table S2: RRs of self-reported “very good” or “excellent” health of increasing quartiles of percent vegetation within a 1000 m radius and a 300 m radius of the reported nearest cross-street adjusted for different combinations of air pollutants, Table S3: Coefficients (and 95% CI) of continuous self-reported health (with higher values indicating better health) of increasing quartiles of percent vegetation from linear models, Table S4: RRs and 95% CIs for the association between % trees, grass and total vegetation as a continuous measure by buffer size with self-reported very good or excellent health compared to good, fair, or poor self-reported health, Table S5: RRs of self-reported very good or excellent health of increasing quartiles of percent trees, grass, or vegetation within various radii of the reported nearest cross-street, Table S6: RRs of self-reported very good or excellent health of increasing quartiles of percent trees, grass, or vegetation within various radii of the reported nearest cross-street also adjusted for NO_2_ at either the 300 m or 1000 m buffer size, whichever is closer in size to the radial buffer for vegetation.
