# Supplementary material for: Is All Urban Green Space the Same? A Comparison of the Health Benefits of Trees and Grass in New York City
Source: Int J Environ Res Public Health. 2017 Nov 18;14(11):1411. doi: 10.3390/ijerph14111411 (PMC5708050; doi:10.3390/ijerph14111411)
Supplement: Supplementary file 1 [file ijerph-14-01411-s001.pdf]

Supplemental Material

Contents

Table S1. Comparison of survey sample retained in analysis to those omitted due to non-geocodeable residential cross-streets 2

Table S2. RRs of self-reported "very good" or "excellent" health of increasing quartiles of percent vegetation within a 1000 m radius and a 300 m radius of the reported nearest cross-street adjusted for different combinations of air pollutants 3

Table S3. Coefficients (and 95% CI) of continuous self-reported health (with higher values indicating better health) of increasing quartiles of percent vegetation from linear models 4

Table S4. RRs and 95% CIs for the association between % trees, grass and total vegetation as a continuous measure by buffer size with self-reported "very good" or "excellent" health compared to "good", "fair", or "poor" self-reported health. 5

Table S5. RRs of self-reported very good or excellent health of increasing quartiles of percent trees, grass or vegetation within various radii of the reported nearest cross-street 6

Table S6. RRs of self-reported very good or excellent health of increasing quartiles of percent trees, grass or vegetation within various radii of the reported nearest cross-street also adjusted for NO_2 at either the 300m or 1000m buffer size, whichever is closer in size to the radial buffer for vegetation 7

Figure S1. Map of tree and grass coverage at 3 ft resolution for all of NYC 8

Figure S2. RRs for self-reported very good or excellent health by quartiles of % grass or % trees at 1000m buffer by categorical SES levels additionally adjusted for individual-level and area-level SES, and NO_2 9

Table S1. Comparison of survey samples retained in analysis to those omitted due to non-geocodeable residential cross-streets.

Variable	Sample Kept (N = 1281)	Omitted from Analysis (N = 278) [a]	*p*-Value [b]
Sex (categorical)	63.6% female	64.3% female	0.80
Education (categorical)	Median = 6 (Associate's degree)	Median = 5 (some college, no degree)	<0.001
Income (categorical)	Median = 3 ($46,000 to just under $70,000)	Median = 2 ($23,000 to just under $46,000)	0.10
Age (continuous)	Mean = 44.7	Mean = 46.4	0.2
How long lived in neighborhood (categorical)	Median = 4 (10 years or more)	Median = 4 (10 years or more)	0.3
Study Arm (categorical)	9.5 % landline<br>32.0% cell phone<br>58.5% online	15.5 % landline<br>46.4% cell phone<br>38.1% online	<0.001
Season (categorical)	48.6% season 1	56.8% season 1	0.02

[a] Number of observations for those dropped from analysis varies by variable and some were dropped due to missing values, [b] *p*-value from χ^2 test for categorical variables and t-test for continuous variables.

Table S2. RRs of self-reported "very good" or "excellent" health of increasing quartiles of percent vegetation within a 1000 m radius and a 300 m radius of the reported nearest cross-street adjusted for different combinations of air pollutants.

Buffer	Exposure	Covariates	First Quartile (Reference)	Second Quartile	Third Quartile	Fourth Quartile
1000 m	Trees	SES + NO_2	1	1.24 (1.05, 1.46) *	1.17 (0.98, 1.39)	1.37 (1.15, 1.63) *
		SES + $PM_{2.5}$	1	1.24 (1.05, 1.45) *	1.17 (0.98, 1.39)	1.34 (1.13, 1.59) *
		SES + NO_2+ $PM_{2.5}$	1	1.24 (1.05, 1.46) *	1.17 (0.98, 1.39)	1.36 (1.15, 1.62) *
		SES + population density	1	1.19 (1.01, 1.40) *	1.10 (0.93, 1.30)	1.25 (1.07, 1.46) *
		SES + NO_2 + population density	1	1.22 (1.03, 1.44) *	1.15 (0.96, 1.37)	1.34 (1.13, 1.60) *
	Grass	SES + NO_2	1	1.09 (0.91, 1.30)	1.23 (1.00, 1.50) *	1.25 (1.00, 1.57) *
		SES + $PM_{2.5}$	1	1.08 (0.91, 1.29)	1.21 (1.00, 1.47) *	1.23 (0.99, 1.52)
		SES + NO_2+ $PM_{2.5}$	1	1.09 (0.91, 1.30)	1.23 (1.00, 1.50) *	1.25 (1.00, 1.57) *
		SES + population density	1	1.02 (0.87, 1.20)	1.13 (0.96, 1.34)	1.14 (0.95, 1.37)
		SES + NO_2 + population density	1	1.08 (0.91, 1.29)	1.24 (1.01, 1.52) *	1.30 (1.03, 1.63) *
	Total Vegetation	SES + NO_2	1	1.09 (0.91, 1.31)	1.27 (1.07, 1.52) *	1.42 (1.15, 1.74) *
		SES + $PM_{2.5}$	1	1.09 (0.91, 1.32)	1.26 (1.06, 1.50) *	1.36 (1.12, 1.67) *
		SES + NO_2+ $PM_{2.5}$	1	1.10 (0.91, 1.32)	1.28 (1.07, 1.52) *	1.41 (1.15, 1.74) *
		SES + population density	1	1.03 (0.86, 1.22)	1.14 (0.98, 1.33)	1.22 (1.04, 1.44) *
		SES + NO_2 + population density	1	1.09 (0.90, 1.31)	1.25 (1.05, 1.49)*	1.41 (1.15, 1.74) *
300 m	Trees	SES + NO_2	1	0.99 (0.83, 1.17)	1.10 (0.94, 1.29)	1.05 (0.88, 1.26)
		SES + $PM_{2.5}$	1	0.99 (0.83, 1.17)	1.11 (0.95, 1.30)	1.04 (0.88, 1.24)
		SES + NO_2+ $PM_{2.5}$	1	0.99 (0.83, 1.17)	1.11 (0.94, 1.30)	1.04 (0.87, 1.24)
		SES + population density	1	0.95 (0.80, 1.13)	1.06 (0.91, 1.23)	0.99 (0.84, 1.17)
		SES + NO2 + population density	1	0.96 (0.81, 1.15)	1.08 (0.92, 1.27)	1.03 (0.86, 1.23)
	Grass	SES + NO_2	1	0.84 (0.70, 1.01)	0.85 (0.71, 1.03)	0.98 (0.80, 1.22)
		SES + $PM_{2.5}$	1	0.85 (0.71, 1.02)	0.86 (0.72, 1.04)	1.00 (0.81, 1.22)
		SES + NO_2+ $PM_{2.5}$	1	0.85 (0.71, 1.02)	0.86 (0.71, 1.04)	0.99 (0.80, 1.23)
		SES + population density	1	0.85 (0.71, 1.01)	0.86 (0.72, 1.02)	1.00 (0.83, 1.20)
		SES + NO_2 + population density	1	0.86 (0.72, 1.03)	0.88 (0.72, 1.07)	1.03 (0.82, 1.29)
	Total Vegetation	SES + NO_2	1	0.89 (0.75, 1.06)	0.98 (0.82, 1.18)	1.01 (0.82, 1.24)
		SES + $PM_{2.5}$	1	0.90 (0.76, 1.07)	0.99 (0.83, 1.17)	1.00 (0.82, 1.22)
		SES + NO_2+ $PM_{2.5}$	1	0.90 (0.76, 1.07)	0.99 (0.82, 1.18)	1.00 (0.81, 1.23)
		SES + population density	1	0.87 (0.74, 1.03)	0.95 (0.81, 1.12)	0.97 (0.82, 1.16)
		SES + NO_2 + population density	1	0.88 (0.75, 1.05)	0.98 (0.82, 1.17)	1.01 (0.82, 1.24)

All models additionally controlled for season, sampling frame, neighborhood tenure, age, sex, race/ethnicity, individual SES (two-level categorical variable for education, and a six-level categorical variable for income), and area-level SES (% of census tract unemployed and % of census tract living below twice the FPL). * $p < 0.05$, SES: socio-economic status.

Table S3. Coefficients (and 95% CI) of continuous self-reported health (with higher values indicating better health) of increasing quartiles of percent vegetation from linear models.

Buffer	Exposure	Covariates	First Quartile (Reference)	Second Quartile	Third Quartile	Fourth Quartile
1000 m	Trees	SES	0	0.15 (0.01, 0.30) *	0.04 (−0.09, 0.18)	0.20 (0.07, 0.33) *
		SES+NO_2	0	0.17 (0.02, 0.31)	0.08 (−0.06, 0.22)	0.26 (0.11, 0.40) *
	Grass	SES	0	0.01 (−0.14, 0.16)	0.08 (−0.08, 0.24)	0.06 (−0.08, 0.20)
		SES+NO_2	0	0.08 (−0.08, 0.24)	0.18 (0.00, 0.36)	0.21 (0.01, 0.41) *
	Total Vegetation	SES	0	0.03 (−0.11, 0.17)	0.06 (−0.09, 0.21)	0.13 (−0.01, 0.27)
		SES+NO_2	0	0.09 (−0.06, 0.25)	0.14 (−0.02, 0.31)	0.28 (0.1, 0.46) *
300 m	Trees	SES	0	0.00 (−0.14, 0.15)	0.07 (−0.07, 0.21)	−0.03 (−0.18, 0.12)
		SES+NO_2	0	0.02 (−0.13, 0.16)	0.09 (−0.06, 0.24)	0.00 (−0.16, 0.16)
	Grass	SES	0	−0.17 (−0.31, −0.02) *	−0.15 (−0.29, 0.00)	−0.08 (−0.23, 0.06)
		SES+NO_2	0	−0.16 (−0.32, 0.00)	−0.14 (−0.31, 0.04)	−0.07 (−0.28, 0.14)
	Total Vegetation	SES	0	−0.05 (−0.20, 0.09)	−0.06 (−0.21, 0.09)	−0.04 (−0.19, 0.11)
		SES+NO_2	0	−0.03 (−0.19, 0.12)	−0.02 (−0.19, 0.15)	0.02 (−0.16, 0.21)

All models additionally controlled for season, sampling frame, neighborhood tenure, age, sex, race/ethnicity, individual SES (two-level categorical variable for education, and a six-level categorical variable for income), and area-level SES (% of census tract unemployed and % of census tract living below twice the FPL). * $p < 0.05$.

Table S4. RRs and 95% CIs for the association between % trees, grass, and total vegetation as a continuous measure by buffer size with self-reported "very good" or "excellent" health compared to "good", "fair", or "poor" self-reported health.

**Buffer**	**Exposure**	**Covariates**	**RR (95% CI)**
1000 m	Trees	SES	1.004 (0.997, 1.011)
		SES + NO_2	1.010 (1.001, 1.018) *
	Grass	SES	1.001 (0.992, 1.009)
		SES + NO_2	1.012 (1.001, 1.024) *
	Total Vegetation	SES	1.002 (0.997, 1.006)
		SES + NO_2	1.009 (1.003, 1.016) *
300 m	Trees	SES	0.998 (0.991, 1.005)
		SES + NO_2	1.002 (0.994, 1.010)
	Grass	SES	0.997 (0.989, 1.005)
		SES + NO_2	1.003 (0.993, 1.014)
	Total Vegetation	SES	0.998 (0.994, 1.003)
		SES + NO_2	1.002 (0.996, 1.008)

All models additionally controlled for season, sampling frame, neighborhood tenure, age, sex, race/ethnicity, individual SES (two-level categorical variable for education, and a six-level categorical variable for income), and area-level SES (% of census tract unemployed and % of census tract living below twice the FPL). * $p < 0.05$.

Table S5. RRs of self-reported "very good" or "excellent" health of increasing quartiles of percent trees, grass, or vegetation within various radii of the reported nearest cross-street.

Exposure	Buffer Size	First Quartile (Reference)	Second Quartile	Third Quartile	Fourth Quartile
Trees	100 m	1	1.06 (0.90, 1.24)	0.95 (0.81, 1.11)	0.95 (0.81, 1.12)
	300 m	1	0.96 (0.81, 1.14)	1.05 (0.91, 1.22)	0.98 (0.83, 1.15)
	500 m	1	1.06 (0.90, 1.25)	1.07 (0.91, 1.26)	1.06 (0.90, 1.25)
	1000 m	1	1.19 (1.01, 1.40) *	1.09 (0.93, 1.29)	1.23 (1.05, 1.43) *
	2000 m	1	1.01 (0.85, 1.20)	1.08 (0.92, 1.26)	1.15 (0.99, 1.34)
Grass	100 m	1	0.89 (0.76, 1.03)	0.81 (0.69, 0.95) *	0.91 (0.78, 1.06)
	300 m	1	0.81 (0.69, 0.95) *	0.81 (0.69, 0.94) *	0.90 (0.78, 1.05)
	500 m	1	0.84 (0.71, 0.99) *	0.90 (0.77, 1.06)	0.96 (0.83, 1.11)
	1000 m	1	0.98 (0.83, 1.16)	1.04 (0.89, 1.22)	1.00 (0.86, 1.17)
	2000 m	1	1.04 (0.88, 1.22)	1.03 (0.87, 1.21)	1.00 (0.85, 1.17)
Total Vegetation	100 m	1	0.82 (0.70, 0.96)	0.86 (0.73, 1.00)	0.90 (0.77, 1.04)
	300 m	1	0.86 (0.73, 1.02)	0.92 (0.79, 1.07)	0.91 (0.77, 1.06)
	500 m	1	1.01 (0.86, 1.20)	0.98 (0.84, 1.16)	1.04 (0.89, 1.22)
	1000 m	1	0.99 (0.83, 1.17)	1.10 (0.95, 1.29)	1.11 (0.96, 1.29)
	2000 m	1	1.09 (0.93, 1.28)	1.09 (0.93, 1.29)	1.18 (1.01, 1.38) *

All models additionally controlled for season, sampling frame, neighborhood tenure, age, sex, race/ethnicity, individual SES (two-level categorical variable for education, and a six-level categorical variable for income), and area-level SES (% of census tract unemployed and % of census tract living below twice the FPL). * $p < 0.05$.

Table S6. RRs of self-reported "very good" or "excellent" health of increasing quartiles of percent trees, grass, or vegetation within various radii of the reported nearest cross-street also adjusted for NO_2 at either the 300 m or 1000 m buffer size, whichever is closer in size to the radial buffer for vegetation.

Exposure	Buffer Size	First Quartile (Reference)	Second Quartile	Third Quartile	Fourth Quartile
Trees	100 m	1	1.07 (0.91, 1.25)	0.97 (0.83, 1.14)	1.00 (0.84, 1.19)
	300 m	1	0.98 (0.83, 1.17)	1.10 (0.94, 1.29)	1.06 (0.89, 1.26)
	500 m	1	1.09 (0.93, 1.28)	1.12 (0.95, 1.32)	1.15 (0.97, 1.37)
	1000 m	1	1.23 (1.04, 1.44) *	1.15 (0.97, 1.37)	1.36 (1.14, 1.61) *
	2000 m	1	1.03 (0.87, 1.23)	1.12 (0.96, 1.31)	1.27 (1.08, 1.50) *
Grass	100 m	1	0.91 (0.77, 1.08)	0.85 (0.71, 1.02)	0.97 (0.79, 1.19)
	300 m	1	0.84 (0.70, 1.00)	0.85 (0.70, 1.03)	0.97 (0.79, 1.19)
	500 m	1	0.90 (0.75, 1.08)	1.01 (0.83, 1.22)	1.11 (0.90, 1.37)
	1000 m	1	1.07 (0.90, 1.28)	1.21 (0.99, 1.48)	1.24 (0.99, 1.55)
	2000 m	1	1.12 (0.94, 1.34)	1.16 (0.95, 1.41)	1.19 (0.95, 1.48)
Total Vegetation	100 m	1	0.83 (0.71, 0.98)	0.90 (0.76, 1.07)	0.97 (0.81, 1.16)
	300 m	1	0.89 (0.75, 1.06)	0.98 (0.82, 1.17)	1.01 (0.82, 1.24)
	500 m	1	1.08 (0.91, 1.29)	1.10 (0.91, 1.32)	1.24 (1.01, 1.53) *
	1000 m	1	1.08 (0.90, 1.30)	1.26 (1.05, 1.50) *	1.40 (1.14, 1.72) *
	2000 m	1	1.16 (0.98, 1.38)	1.22 (1.02, 1.46) *	1.46 (1.19, 1.79) *

All models additionally controlled for season, sampling frame, neighborhood tenure, age, sex, race/ethnicity, individual SES (two-level categorical variable for education, and a six-level categorical variable for income), and area-level SES (% of census tract unemployed and % of census tract living below twice the FPL). * $p < 0.05$.

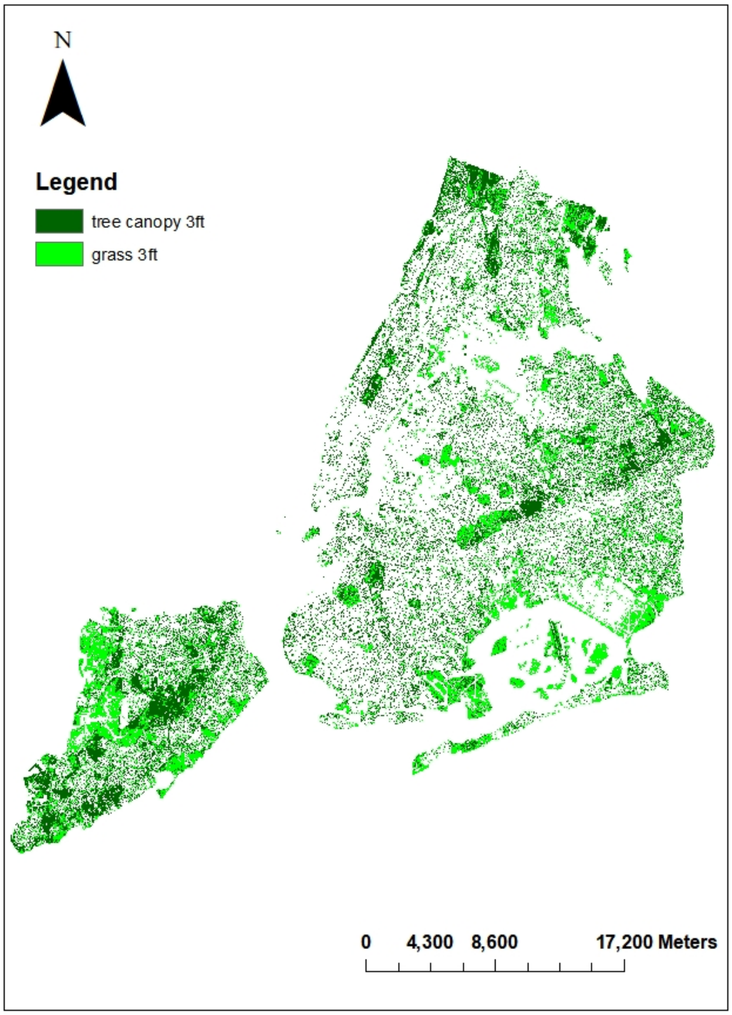

Figure S1. Map of tree and grass coverage at 3-ft resolution for all of NYC. NYC = New York City.

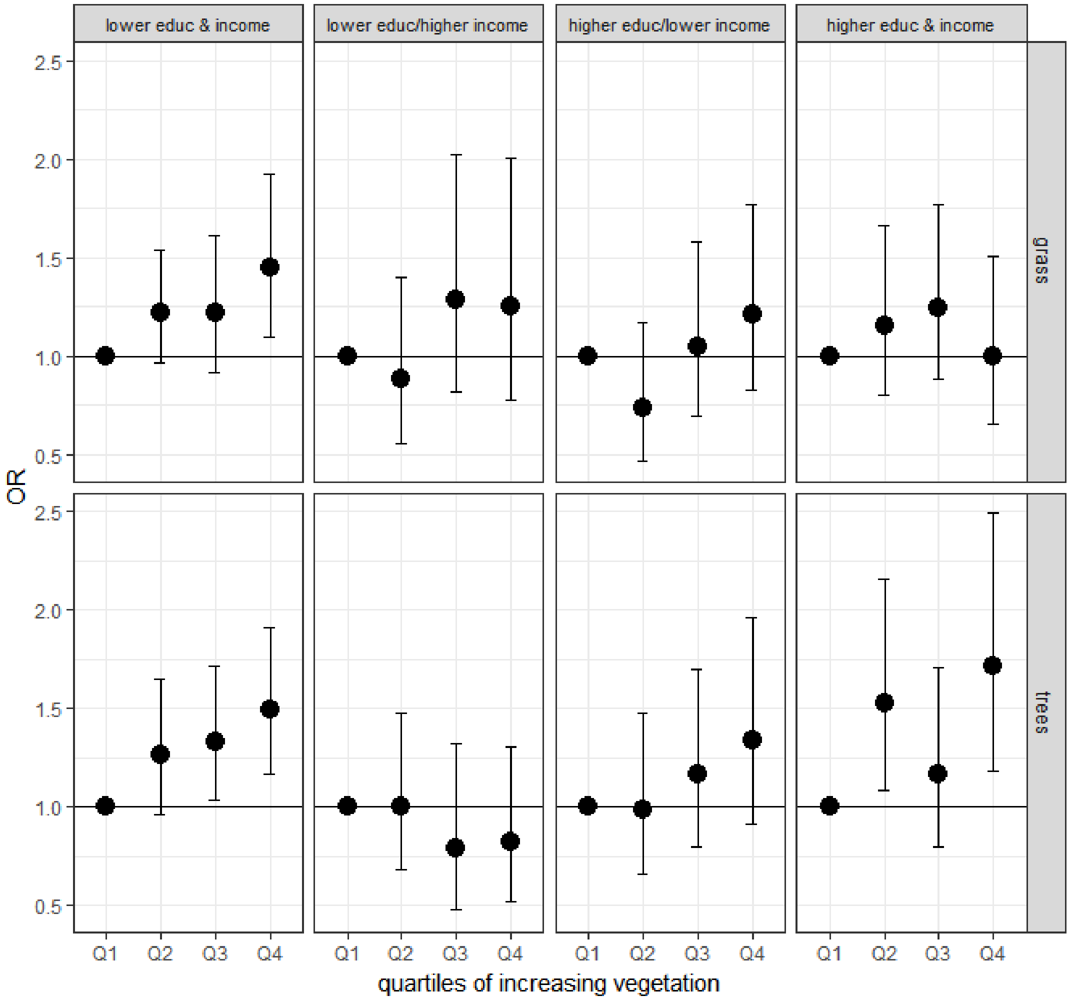

Figure S2. RRs for self-reported "very good" or "excellent" health by quartiles of % grass or % trees at 1000 m buffer by categorical SES levels additionally adjusted for individual-level and area-level SES and NO_2.
